# Sex-specific and opposite modulatory aspects revealed by PPI network and pathway analysis of ischemic stroke in humans

**DOI:** 10.1371/journal.pone.0227481

**Published:** 2020-01-03

**Authors:** Yan Lv, XY He, Dongguo Li, Tao Liu, GQ Wen, Junfa Li

**Affiliations:** 1 Department of Neurobiology, School of Basic Medical Sciences, Capital Medical University, Beijing, China; 2 Department of Neurology, Hainan General Hospital, Haikou, China; 3 Department of Bioinformatics and Engineering, School of Basic Medical Sciences, Capital Medical University, Peking, China; Universiti Tunku Abdul Rahman, MALAYSIA

## Abstract

**Background:**

Ischemic Stroke (IS) is a major disease which greatly threatens human health. Recent studies showed sex-specific outcomes and mechanisms of cerebral ischemic stroke. This study aimed to identify the key changes of gene expression between male and female IS in humans.

**Methods:**

Gene expression dataset GSE22255, including peripheral blood samples, was downloaded from the Gene Expression Omnibus (GEO) dataset. Differentially Expressed Genes (DEGs) with a LogFC>1, and a P-value <0.05 were screened by BioConductor R package and grouped in female, male and overlap DEGs for further bioinformatic analysis. Gene Ontology (GO) functional annotation, Protein-Protein Interaction (PPI) network, “Molecular Complex Detection” (MCODE) modules, CytoNCA (cytoscape network centrality analysis) essential genes and Kyoto Encyclopedia of Genes and Genomes (KEGG) pathway interrelation analysis were performed.

**Results:**

In a total of 54,665 genes, 185 (73 ups and 112 downs) DEGs in the female dataset, 461 DEGs (297 ups and 164 downs) in the male dataset, within which 118 DEGs overlapped (7 similar changes in female and male, 111 opposite changes in female and male) were obtained from the GSE22255 dataset. Female, male and overlapping DEGs enriched for similar cellular components and molecular function. Male DEGs enriched for divergent biological processes from female and overlapping DEGs. Sex-specific and overlapping DEGs were put into the PPI network. Overlapping genes such as IL6, presented opposite changes and were mainly involved in cytokine-cytokine receptor interactions, the TNF-signalling pathway, etc.

**Conclusion:**

The analysis of sex-specific DEGs from GEO human blood samples showed that not only specific but also opposite DEG alterations in the female and male stroke genome wide dataset. The results provided an overview of sex-specific mechanisms, which might provide insight into stroke and its biomarkers and lead to sex-specific prognosis and treatment strategies in future clinical practice.

## 1.Introduction

Stroke is the leading cause of disability and the second leading cause of death worldwide, with an annual incidence of approximately 17 million. It is estimated that every 40 seconds, there is a stroke in the United States, 87% of which are ischemic strokes [1]. Currently, urgent-in-time thrombosis treatment is an effective therapeutic strategy and requires long-term antiplanet or anticoagulant medication to prevent IS recurrence [2].

Sex differences were seen for IS as females had higher incidence and longer life expectancy but worse functional outcomes [3,4], which might be due to different risk factors [5,6,7], anatomic structural Willis incompletion or white integrity [8,9], biologically inherent sex chromosome complemented with gonadal hormones [10,11,12], socialized reasons of in-hospital care [6,13], and therefore, pathology and treatment [14,15]. However, animal-based research has observed decreased infarct size and improved outcomes in female compared to males [16]. Otherwise, a parallel characterization of the cytokine and chemokine response to stroke in the human and mouse brain at different stages of infarct resolution have been reported [17]. Many studies have observed sex differences in IS; however, the mechanism has yet to be elucidated.

High-throughput platforms for analysis of gene expression, such as microarrays, are promising tools for inferring biological relevancy, especially the complex network during the process of IS. However, there is no genome-wide exploration of the sex-specific mechanism in IS. In this study, we investigated the well-documented original data from the Gene Expression Omnibus (GEO) [18]. The human stroke blood sample dataset, GSE22255, was selected for further exploration [19]. Gene ontology and biological function annotation were performed followed by PPI network and related analysis. By using the bioinformatic method, new insight into the mechanisms of IS can be obtained which may reveal potential biomarker candidates for clinical use and drug target discovery.

## 2. Materials and methods

### 2.1. Microarray data

The authors declare that all supporting data are available within the article [and its online supplementary files]. The GEO dataset mining results showed that few homogeneous studies available (S1 Fig). So, for the study object of translation of clinical practice, we chose human whole blood cell datasets for this comparative bioinformatic analysis work. The gene expression profiles of GSE22255 as whole blood cell datasets for human cerebral ischemia were downloaded from the GEO [20]. GSE22255 was performed on GPL570, [HG-U133 Plus 2] Aymetrix Human Genome U133 Plus2.0 Array. The GSE22255 data set contained 40 samples, including 10 female IS patients, 10 male IS patients, 10 female controls, and 10 male controls [19]. The imported CEL files were subjected to background correction, normalization, and summarization using the robust multichip average (RMA) algorithm for normalization [19]. The GSE22255 study was approved by the ethics committees of the participating institutions. All participants were informed of the study and provided informed consent [19]. For the analysis of this study, no ethics approval and patients’ informed consent was needed.

### 2.2. Identification of DEGs

The analysis was carried out by open source software which significantly simplified the development and distribution of preprocessing methods for gene expression microarrays with BioConductor R and package (https://www.r-project.org/) [21,22]. The expression of the GSE22255 DataSet full SOFT file was downloaded (https://www.ncbi.nlm.nih.gov/sites/GDSbrowser?acc=GDS4521). Female and male IS patients and controls were assigned according to the annotation of the GSE22255. DEGs were identified after probe signal average and normalized, where the log_2_ of a total of 54,665 genes were calculated. The genes with LogFC>1 and a *P*-value <0.05 were considered differentially expressed. Sex-specific DEGs were obtained by comparison of female IS patients with female controls, and male IS patients with male controls, respectively.

### 2.3. Bioinformatic analysis

#### 2.3.1 Gene ontology and pathway enrichment analysis of DEGs

Gene Ontology (GO) [23] cellular component, molecular function, biological process and KEGG pathway enrichment were analysed using a web-based tool, Search Tool for the Retrieval of Interacting Genes (STRING) (version 10.5) (https://string-db.org/) [24]. Enrichment tests were based on the hypergeometric distribution [25]. False discovery rate was used to evaluate significance [26].

#### 2.3.2 Integration of Protein-Protein Interaction (PPI) network analysis

STRING was used to evaluate the interactive (PPI) relationships between DEGs. Evidence-based interactions including text mining, experimental and database interactions achieving combined score >0.4 were selected as significant[24]. PPI networks were constructed using the Cytoscape software [27].

A Cytoscape plug-in, MCODE [28], was used to screen the modules of PPI network identified. Modules inferred using the default settings with degree cutoff at 2, node score cutoff at 0.2, *K* core at 2, and a maximum depth of 100.

A Cytoscape plug-in, CytoNCA [29], which integrated calculation, evaluation, and visualization analysis for multiple centrality, was proposed to screen essential DEGs. PPI network topological structure and relationship characteristics including: Betweenness Centrality (BC), Closeness Centrality (CC), Degree Centrality (DC), Eigenvector Centrality (EC), Local Average Connectivity-based Centrality (LAC), Network Centrality (NC), Subgraph Centrality (SC), and Information Centrality (IC) were calculated. The appropriate minimum threshold varied and was determined by the network nodes and edges distributions. Top 10 essential DEGs were screened.

#### 2.3.3 Pathways enrichment and interrelation analysis

KEGG pathways enrichment and interrelation Analysis. Pathway enrichment analysis was carried out using DAVID(The Database for Annotation, Visualization and Integrated Discovery) databse[30]. Group *P-*values <0.01, minimum gene in clusters was set at 4. Enriched KEGG pathway and overlapped DEGs interrelation analysis for female and male IS patients was conducted and the interrelation network was reconstructed in Cytoscape software.

## 3. Results

The bioinformatic analysis results were following the route showed in S2 Fig.

### 3.1. Identification of DEGs

**Fig 1:**
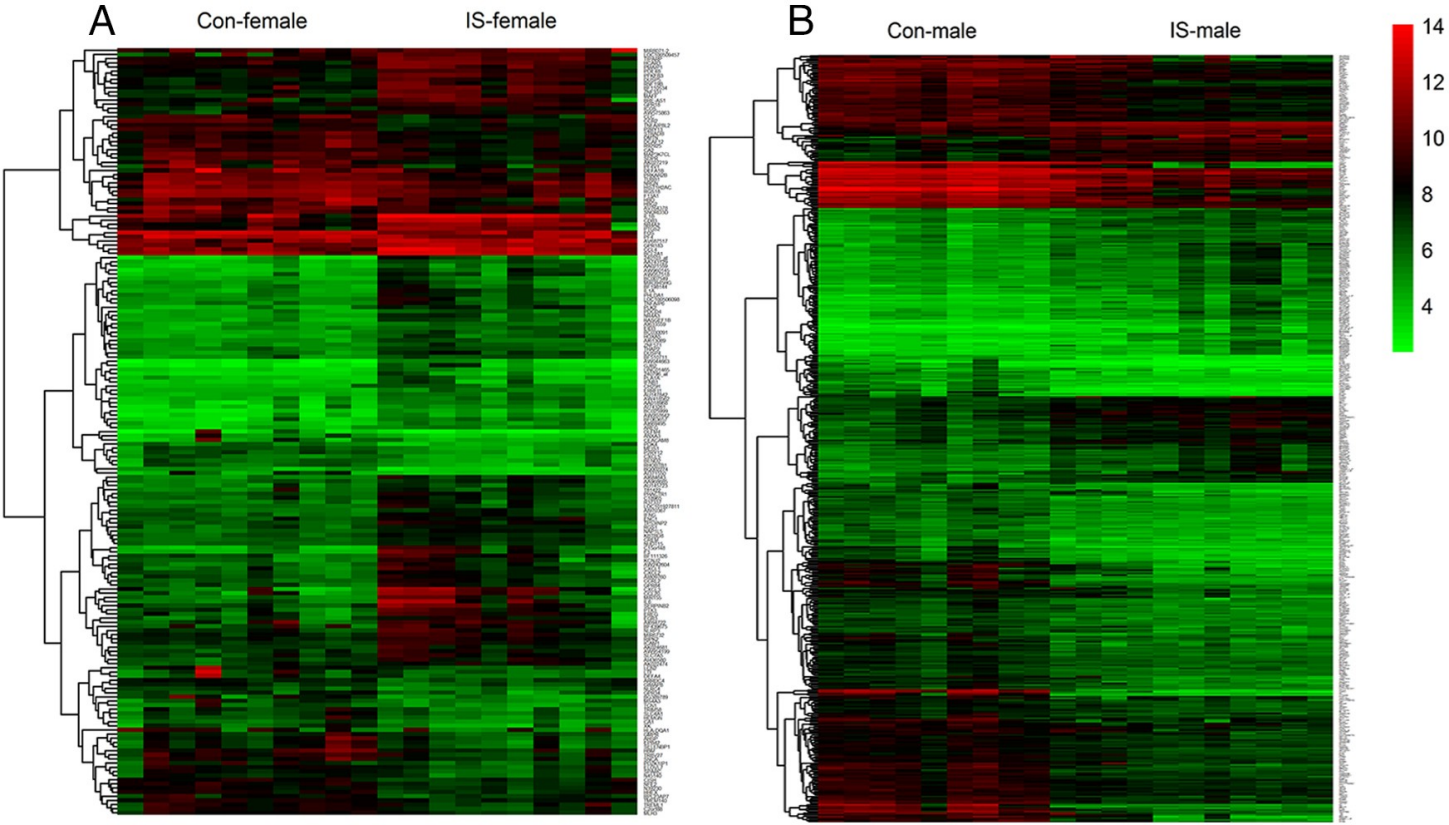
Sex-specific DEGs in IS.

The GSE22255 data set contained 40 peripheral blood samples, including 10 female IS patients, 10 male IS patients, 10 female controls, and 10 male controls. IS patients were required to have suffered only one stroke episode, at least 6 months before the blood collection, and controls could not have a family history of stroke. Participants with severe anemia or active allergies were also excluded [19]. Sex-specific DEGs were obtained by comparison of female IS patients with female controls, and male IS patients with male controls, respectively. All sex-specific DEGs are presented in Fig 1. In the female dataset, 185 (73 upregulated and 112 downregulated) DEGs were obtained, while 461 DEGs (297 upregulated and 164 downregulated), within which 118 DEGs overlapped (7 similar changes and 111 opposite changes in female and male), were obtained in the male dataset.

### 3.2. Bioinformatic analysis

For the 185 female-specific DEGs, 461 male-specific DEGs, 118 overlapping DEGs, functional annotation was carried out. However, only part of the collection obtained functional annotations. The functionally annotated DEGs including 126 (68.1%) female-specific, 250 (54.2%) male-specific, and 75 (63.6%) overlapping DEGs were put for further GO, KEGG and PPI analysis.

#### 3.2.1 GO and pathway enrichment analysis

Top 5 or less enrichment analyses results were shown for each part of the GO analysis. The cellular component, molecular function and biological process enrichment analyses results are shown in Table 1. Female, male and overlapping DEGs significantly took part in similar and overlapping cellular components, molecular functions and KEGG pathways, but divergent biological processes. For cellular component analysis, female, male and overlapping DEGs mainly enriched in the extracellular space (GO.0005615). For molecular function, female, male and overlapping DEGs mainly enriched in chemokine receptor binding (GO.0042379) and cytokine activity (GO.0005125). For GO biological process enrichment analysis, male-specific DEGs took part in regulation of apoptotic processes (GO.0042981), regulation of cell death (GO.0010941), positive regulation of cell death (GO.0010942), and positive regulation of programmed cell death (GO.0043068).

**Table 1:** GO enrichment analysis in networks.

GO	gender	pathway ID	pathway description	observed gene count	false discovery rate
CC	female	GO.0005615	extracellular space	34	4.10E-11
		GO.0005833	haemoglobin complex	4	0.000138
		GO.0044421	extracellular region part	42	0.00292
		GO.0030141	secretory granule	10	0.00505
	male	GO.0005615	extracellular space	35	0.00229
	overlap	GO.0005615	extracellular space	17	0.00223
		GO.0009897	external side of plasma membrane	7	0.0132
		GO.0098552	side of membrane	8	0.0484
MF	female	GO.0042379	chemokine receptor binding	10	1.88E-09
		GO.0005125	cytokine activity	13	1.39E-08
		GO.0005126	cytokine receptor binding	14	4.65E-08
		GO.0008009	chemokine activity	8	2.06E-07
		GO.0045236	CXCR chemokine receptor binding	6	3.03E-07
	male	GO.0005126	cytokine receptor binding	20	3.86E-09
		GO.0005125	cytokine activity	15	7.71E-07
		GO.0042379	chemokine receptor binding	9	1.17E-05
		GO.0005102	receptor binding	34	0.000166
		GO.0000982	transcription factor activity, RNA polymerase II core promoter proximal region sequence-specific binding	17	0.000243
	overlap	GO.0005125	cytokine activity	10	5.22E-07
		GO.0005126	cytokine receptor binding	11	5.22E-07
		GO.0042379	chemokine receptor binding	7	8.87E-07
		GO.0008009	chemokine activity	5	0.000549
		GO.0005102	receptor binding	16	0.000865
BP	female	GO.0009617	response to bacterium	25	1.85E-13
		GO.0051707	response to other organisms	27	4.89E-12
		GO.0032496	response to lipopolysaccharide	19	8.24E-12
		GO.0006955	immune response	35	1.12E-11
		GO.0033993	response to lipid	27	7.12E-11
	male	GO.0042981	regulation of apoptotic process	59	1.45E-15
		GO.0010941	regulation of cell death	59	1.19E-14
		GO.0002237	response to molecule of bacterial origin	28	1.53E-14
		GO.0010942	positive regulation of cell death	37	5.69E-14
		GO.0043068	positive regulation of programmed cell death	36	5.69E-14
	overlap	GO.0006954	inflammatory response	16	1.80E-08
		GO.0002684	positive regulation of immune system process	20	2.03E-08
		GO.0032496	response to lipopolysaccharide	13	3.93E-08
		GO.0033993	response to lipid	19	3.93E-08
		GO.0060326	cell chemotaxis	11	3.93E-08

CC: cellular component; MF: molecular function; BP: biological process

#### 3.2.2. PPI network and module screening

**Fig 2:**
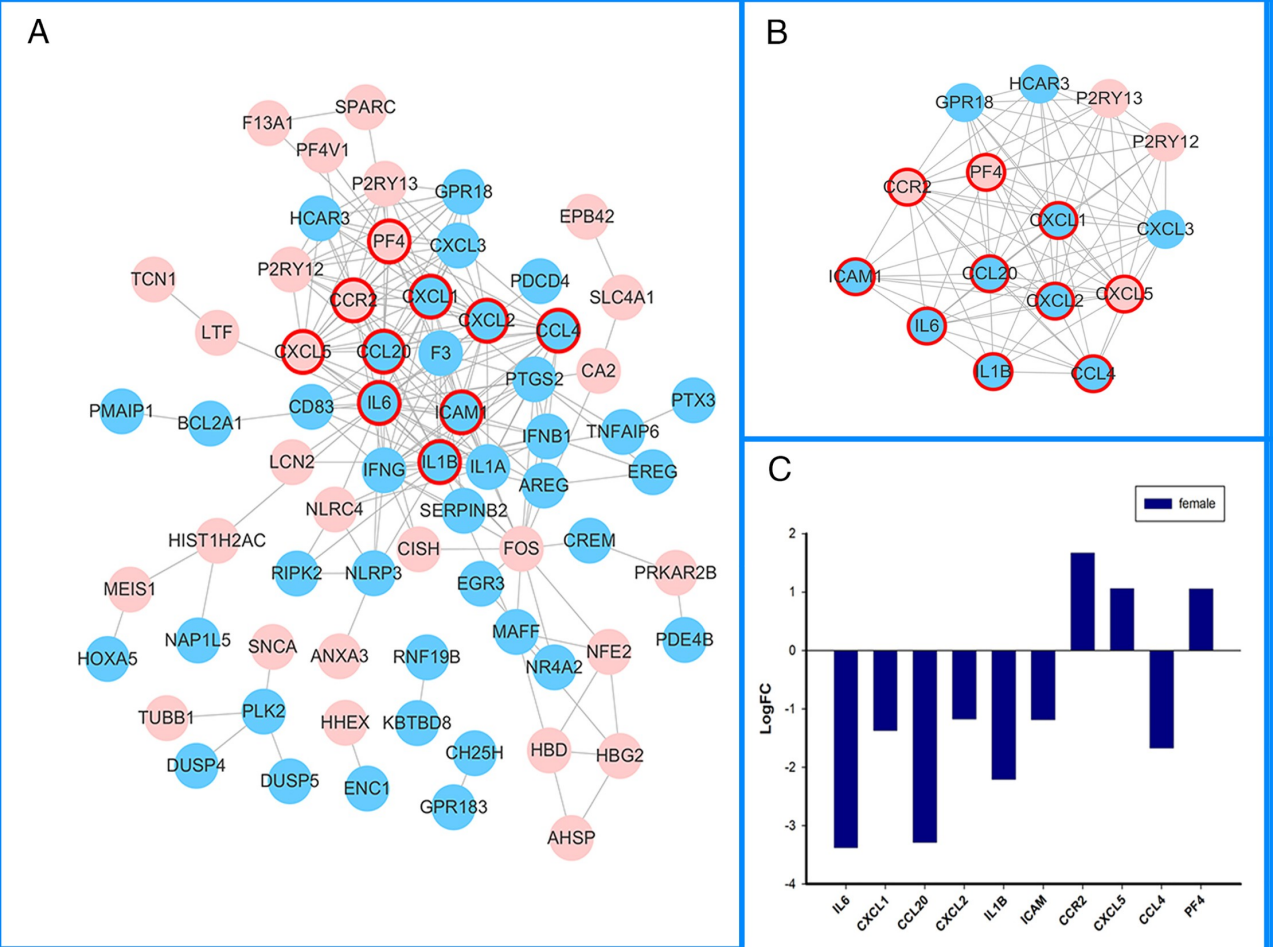
Female-specific PPI networks in IS. A. Pink circles represent upregulated DEGs, blue circles represent downregulated DEGs, thicker red border emphasizes CytoNCA topologically significant DEGs. B. MCODE cluster of PPI networks. C. Top 10 CytoNCA significant DEGs. The isolated DEGs indicated no experimentally validated or predicted protein-protein interactions.

**Fig 3:**
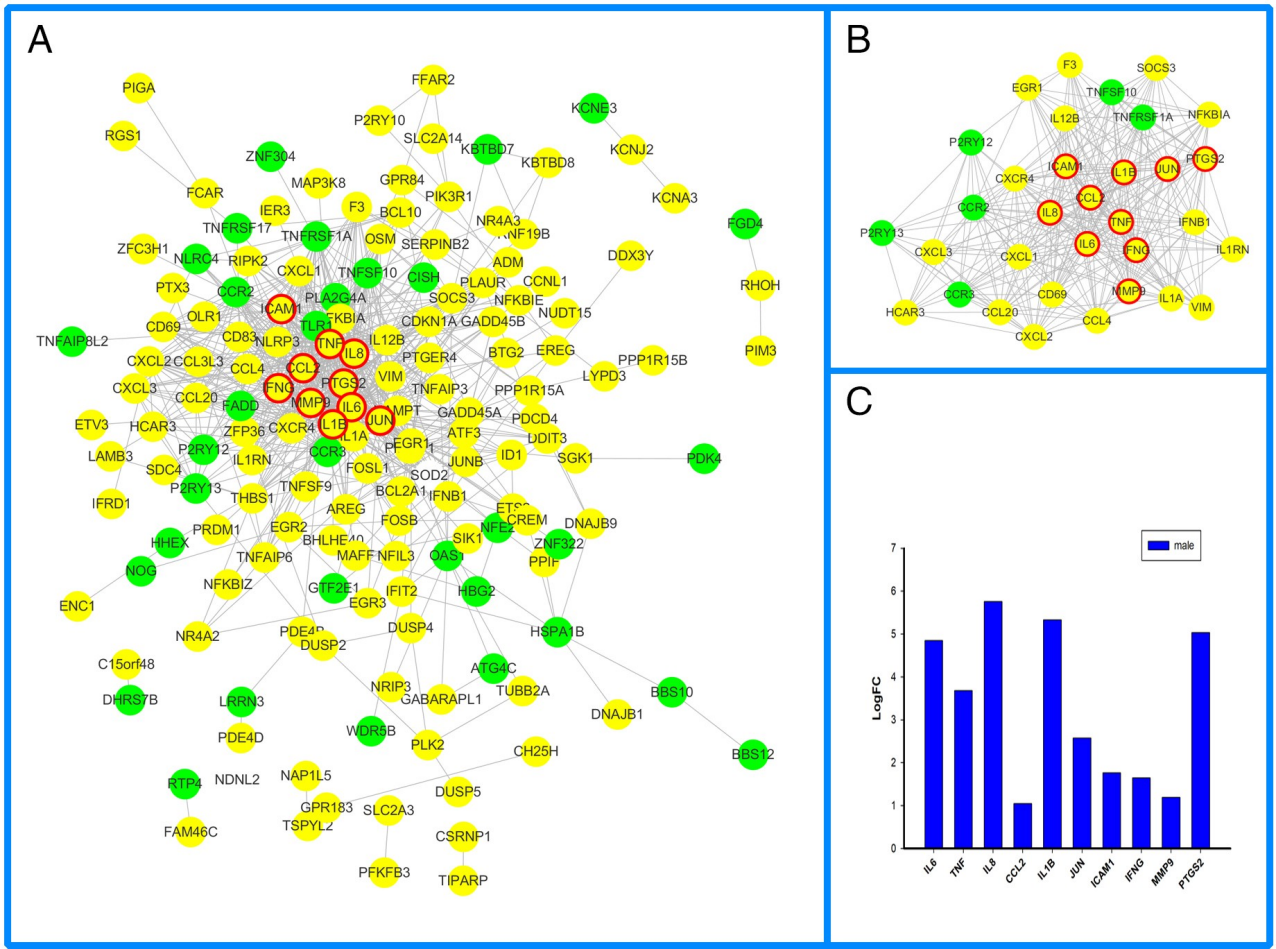
Male-specific PPI network sin IS. Yellow circles represent upregulated DEGs, green circles represent downregulated DEGs, thicker red border emphasizes CytoNCA topologically significant DEGs. B. MCODE cluster of PPI networks. C. Top 10 CytoNCA significant DEGs. The isolated DEGs indicated no experimentally validated or predicted protein-protein interactions.

**Fig 4:**
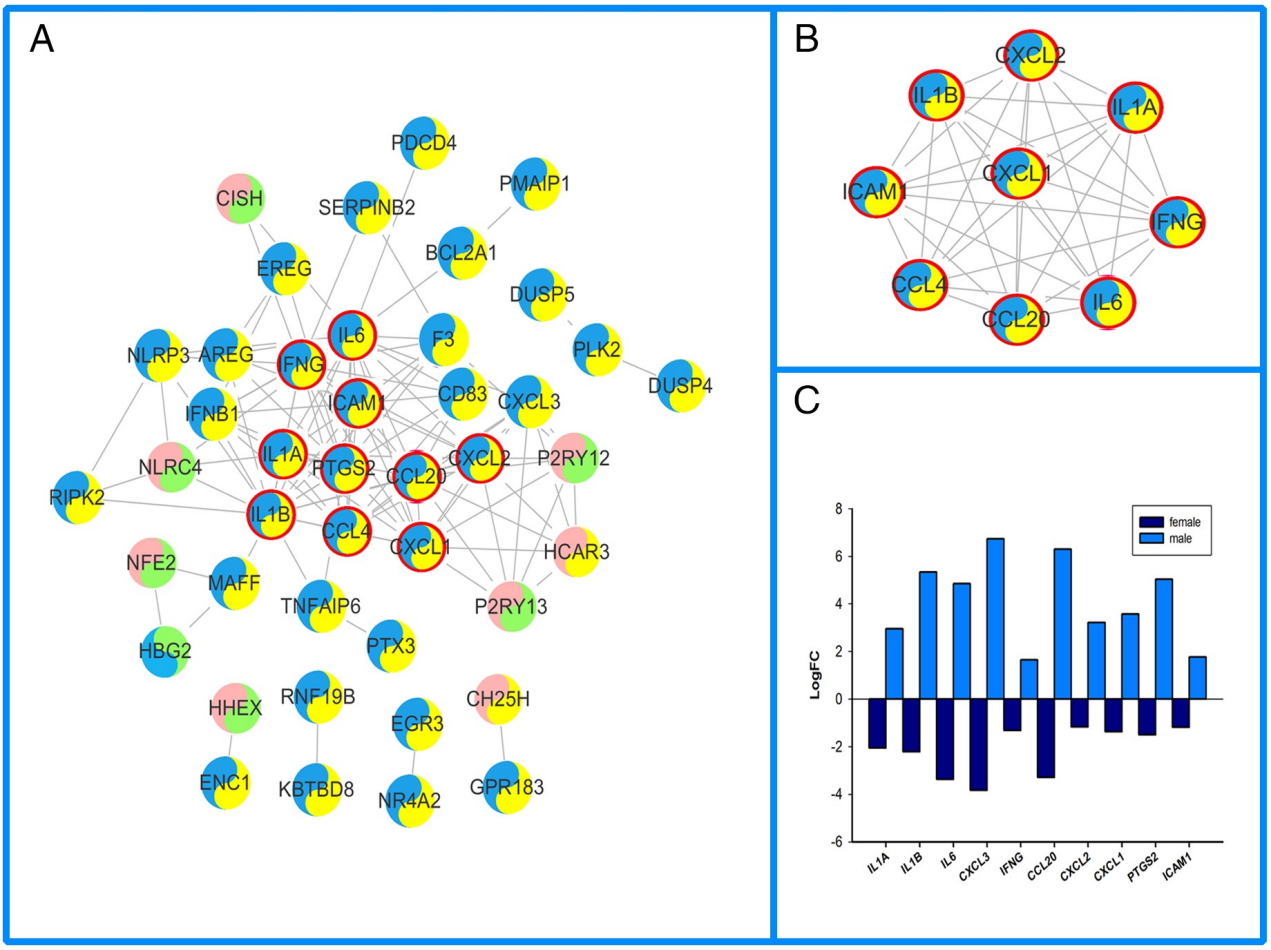
Female and male overlapping PPI networks in IS. A. Coregulated DEGs were shown in a tow color circle. Pink represents upregulated DEGs in females, blue represents downregulated DEGs in females, yellow stands upregulated DEGs in males, green represents downregulated DEGs in males; thicker red border emphasizes CytoNCA topologically significant DEGs. B. MCODE cluster of PPI networks. C. Top 10 CytoNCA significant DEGs.

Based on the information in the STRING database, female- (Fig 2A), male-specific (Fig 3A) and overlapping (Fig 4A) DEGs were put into PPI networks. The PPI networks were analyzed using the Cytoscape plug-in, MCODE. Significant modules were clustered (Fig 2B, Fig 3B, Fig 4B, Table 2). The topologically essential DEGs screened by CytoNCA were outlined by thicker border circles (Fig 2A, Fig 3A, Fig 4A). The top 10 significant fold change in overlapping DEGs were shown in parallel with PPI (Fig 2C, Fig 3C, Fig 4C).

**Table 2:** MCODE modules from PPI networks.

description		MCODE score	Nodes
female	cluster1	11	GPR18 PF4 CCL4 CCR2 CCL20 ICAM1 P2RY13 CXCL1 CXCL2 CXCL5 CXCL3 P2RY13 HCAR3
	cluster2	4.8	IL6 IFNG FOS PTGS2 IL1A IL1B
male	cluster1	11	P2RY13 CCR3 CXCL1 HCAR3 CCR2 IL8 CXCL3 CXCR4 CXCL2 P2RY12 CCL20
	cluster2	6.286	ICAM1 JUN CCL2 TNF IL1B IFNG IL6 PTGS2
overlap	cluster1	7	P2RY13 CCL20 CXCL1 HCAR3 CXCL2 P2RY12 CXCL

#### 3.2.3. KEGG pathway enrichment and interrelation analysis

**Fig 5:**
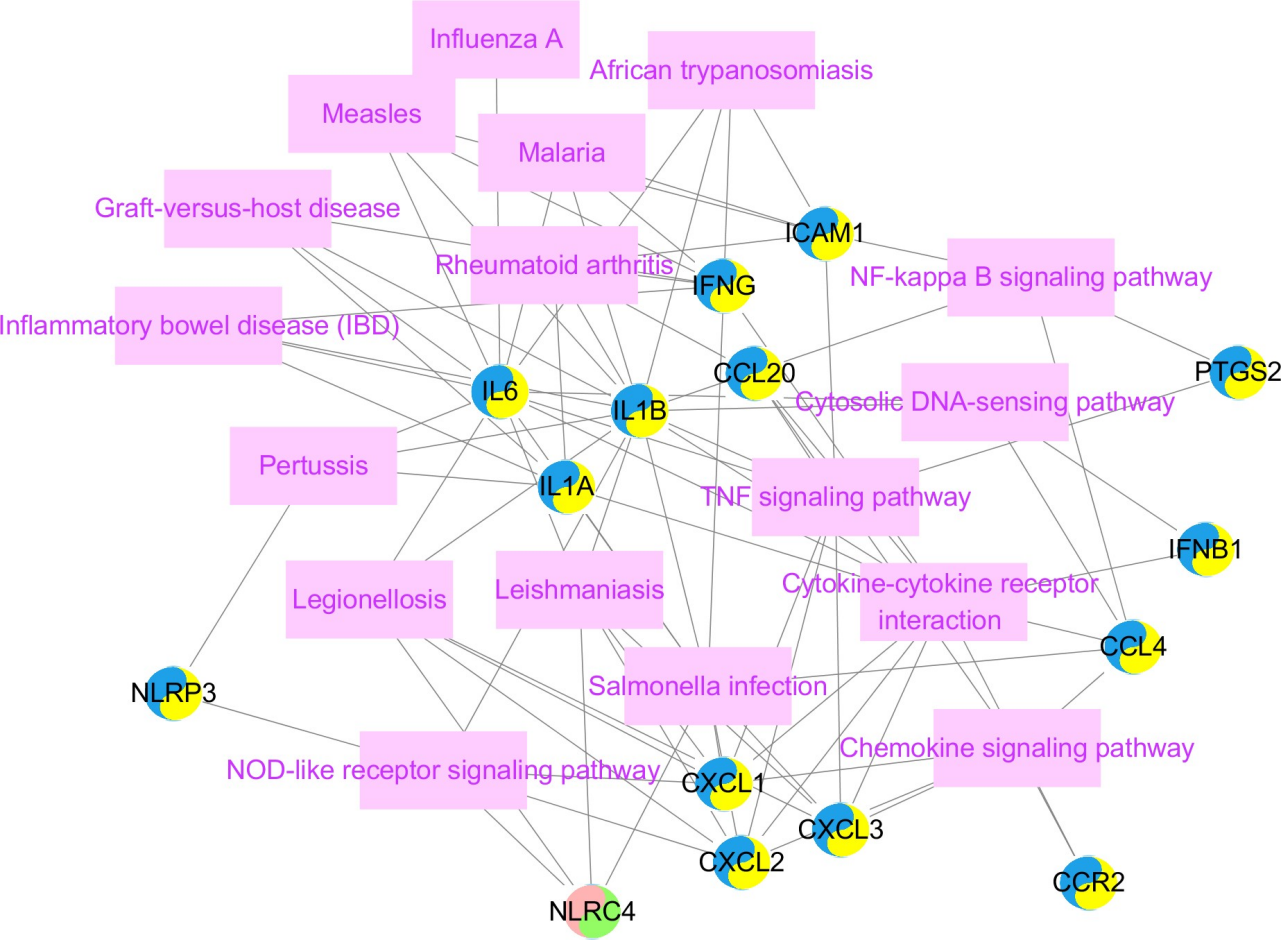
KEGG pathways enrichment and interrelation analysis. Coregulated DEGs were shown in a tow color circle. Pink represents upregulated DEGs in females, blue represents downregulated DEGs in females, yellow stands upregulated DEGs in males, green represents downregulated DEGs in males; pink rectangles represent enriched KEGG pathways. The protein-protein interactions were not included in the pathway and DEG interrelations.

For the female- and male-specific DEGs that presented opposite modulatory mechanisms in overlapping DEGs, the pathway interrelations were used to investigate the bi-modulatory (female-down-male-up) DEGs as shown in Table 3. These genes are mainly involved in pathways including NOD-like receptor signalling pathways, cytokine-cytokine receptor interactions, TNF-signalling pathway, Rheumatoid arthritis, and NF-kappa B-signalling pathway etc (Fig 5). The detailed interactions were included in the S3 Fig_html. Interleukin-1B (IL1B) and Interleukin-6 (IL6) took part in pathways which are the NOD-like receptor signalling pathways, cytokine-cytokine receptor interactions, TNF-signalling pathway, and Rheumatoid arthritis pathways; C-X-C motif chemokine ligands (CXCL) 1, 2, and 3 took part in 2–3 pathways including the TNF-signalling pathway, cytokine-cytokine receptor interactions and NOD-like receptor signalling pathways etc.

**Table 3:** Interrelation between overlapping DEGs and enriched KEGG pathways.

Pathway	gene count	p value	genes
Salmonella infection	9	5.80E-09	CCL4,CXCL1,CXCL2,CXCL3,NLRC4,IFNG,IL1A,IL1B,IL6
Cytokine-cytokine receptor interaction	11	2.10E-07	CCL20,CCL4,CCR2,CXCL1,CXCL2,CXCL3,IFNB1,IFNG,IL1A,IL1B,IL6
TNF signaling pathway	8	8.50E-07	CCL20,CXCL1,CXCL2,CXCL3,ICAM1,IL1B,IL6,PTGS2
Legionellosis	6	6.70E-06	CXCL1,CXCL2,CXCL3,NLRC4,IL1B,IL6
NOD-like receptor signaling pathway	6	8.00E-06	CXCL1,CXCL2,NLRC4,NLRP3,IL1B,IL6,
Rheumatoid arthritis	6	7.30E-05	CCL20,ICAM1,IFNG,IL1A,IL1B,IL6,
Influenza A	7	2.10E-04	NLRP3,ICAM1,IFNB1,IFNG,IL1A,IL1B,IL6
African trypanosomiasis	4	5.90E-04	ICAM1,IFNG,IL1B,IL6
Graft-versus-host disease	4	5.90E-04	IFNG,IL1A,IL1B,IL6
Malaria	4	1.90E-03	ICAM1,IFNG,IL1B,IL6
Chemokine signaling pathway	6	2.30E-03	CCL20,CCL4,CCR2,CXCL1,CXCL2,CXCL3
Cytosolic DNA-sensing pathway	4	4.10E-03	CCL4,IFNB1,IL1B,IL6
Inflammatory bowel disease (IBD)	4	4.10E-03	IFNG,IL1A,IL1B,IL6
Measles	5	4.40E-03	ICAM1,IFNG,IL1B,IL6
Leishmaniasis	4	5.40E-03	CXCL1,CXCL2,CXCL3,NLRC4,IL1B,IL6
Pertussis	4	6.30E-03	NLRP3,IL1A,IL1B,IL6
NF-kappa B signaling pathway	4	9.60E-03	CCL4,ICAM1,IL1B,PTGS2,

## 4. Discussion

Genome-wide analysis (GWAS) has exposed significant aspects of ischemic stroke [31]. Some important SNP polymorphisms were discovered associated with IS including 5 C-reactive protein (CRP) SNPs [32], or (rs9943582, -154G/A) in the 5' flanking region of (APLNR) was shown to be significantly associated with stroke in the Japanese population. In the previous study, 16 genes including TTC7B significantly changed in the IS group compared with age- and gender-matched controls [19]. On the hypothesis that female and male IS had different mechanisms, female and male IS were compared to healthy controls. As the results showed, 185 (73 upregulated and 112 downregulated) DEGs were obtained in the female dataset, 461 DEGs (297 upregulated and 164 downregulated) in the male dataset. In sex-specific DEGs, there were a group of overlapping DEGs but with opposite changes, which lead to a comparatively smaller amount of DEGs in the overall group. The IS blood sample also presented a smaller amount of DEGs compared with early or advanced atherosclerotic plaque samples [33]. The probable reason is that IS is a less stable pathological state and lacks biological markers compared with atherosclerotic plaque. Males double the DEGs and a larger portion of upregulated DEGs than females in this study, which indicated an underlying recovery mechanism for male IS protection and better outcomes than females [3]. After the identification of sex-specific DEGs in IS, the DEGs were put into multi-step bioinformatic functional annotations. The functionally annotated DEGs including 126 (68.1%) female-specific, 250 (54.2%) male-specific, and 75 (63.6%) overlapping DEGs were put for further GO, KEGG and PPI analysis. Although the functional annotation category did not cover all the DEGs, this information could still provide a whole overview and therapeutic drug strategy for sex-specific and opposite modulatory mechanisms in IS patients.

The cross-talks between the vascular and immune system play a critical role in both female and male strokes. Female, male, and overlapping DEGs enriched in the functional categories of chemokine receptor binding (GO.0042379) and cytokine activity (GO.0005125) (Table 1). In this study, IL6 not only obtained key centrality but also gained high centrality in female, male and overlapping PPI networks (Figs 2, 3 and 4). KEGG pathway enrichment and interrelation analysis of female and male overlapping DEGs showed the involved interrelation between the pathways and bi-modulatory (female-down-male-up) DEGs (Table 3, Fig 5). These genes were mainly involved in pathways including NOD-like receptor signalling pathways, cytokine-cytokine receptor interactions, TNF-signalling pathway, Rheumatoid arthritis, and the NF-kappa B-signalling pathway. IL1B took part in 5 pathways, and IL6 took part in 4 pathways which are NOD-like receptor signalling pathways, cytokine-cytokine receptor interactions, TNF-signalling pathway, and Rheumatoid arthritis pathways; CXCL1, CXCL2, and CXCL3 took part in 2–3 pathways including the TNF-signalling pathway, cytokine-cytokine receptor interactions, and NOD-like receptor signalling pathways. Several studies have shown that immune responses including IL6 [34], IgA[35], CXCL16 [36], TNFSF4[37], and IL10 [38] were involved in stroke. IL6 and infarction size were reported as independent predictors of short-term stroke outcome in young Egyptian adults [34]. For the opposite changes of these DEGs, females presented downregulated tendencies for these inflammatory and immune process-related pathways, while males presented the opposite (Fig 3). The divergent immunological alterations were also observed in mice MCAO explorations, where males had a greater percentage of activated macrophages/microglia in the brain than females, as well as increased expression of VLA-4 adhesion molecules in both the brain and spleen [39]. However, microglia (MG) from female mice had higher expression of IL-4 and IL-10 receptors and increased production of IL-4, especially after treatment with IL-10 (+) B-cells, which indicated that females had heightened sensitivity of MG to IL-4 and IL-10 as direct B-cell/MG interactions promote M2-MG [40]. The incongruent results of immune cell participation were related to stroke stage and age. In our study, the increased DEGs, including interleukins and CXCLs, indicated chronic immune activation of cytokine pathways in males might lead to a protective, instead of damaging, process of neuron regeneration.

Another aspect of female and male IS mechanisms would be cross-talk of inflammation and immune processes between apoptosis pathways. For GO biological process enrichment analysis (Table 1), the male-specific DEGs were involved in regulation of the apoptotic process (GO.0042981), regulation of cell death (GO.0010941), positive regulation of cell death (GO.0010942), and positive regulation of programmed cell death (GO.0043068). NF-kappa B-signalling pathway including BCL2A1, CCL4, CXCL2, ICAM1, IL1B, PTGS2, were significantly enriched in male IS patients (Fig 5). Male IS presented upregulated BCL2A1 and CXCL2, while female IS presented downregulated BCL2A1 in this study (Fig 4). The anti-apoptosis protein BCL2 could provide neuron protectiveness via different pathways [41]. Therefore, the apoptosis modulation between females and males would be a cause for different outcomes between them. The mitochondrion is a key factor both in acute and chronic stroke damage recovery [42]. However, since our dataset is based on human blood samples, there would be insufficiency on this platform to screen metabolic processes which mainly occur in the cytoplasm and mitochondrion. The sample size is also another restriction of this study, even though we hope the significant results could raise more attention, so the sex specific mechanism could lead to the modifications of the current clinical practice.

## 5. Conclusion

The analysis of sex-specific DEGs from GEO human blood samples showed not only specific but also opposite DEG alterations in females and males such as IL6, in the stroke genome-wide dataset. The inflammatory immune process and anti-apoptosis pathways presented divergent sex-specific alterations in IS. The results provided an overview of sex-specific mechanisms, which delivered further insight into stroke and potential biomarkers that can lead to sex-specific prognosis and treatment strategies in future clinical practice. However, detailed experimental validation through a combination of *in vitro* and *in vivo* testing is still required.

## Supporting information

S1 Fig GEO datasets screen route. (TIF)

S2 Fig Bioinformatic results route. (TIF)

S3 Fig The detailed interactions of DEGs in enriched pathways. (RAR)

## References

[1] Mozaffarian D, Benjamin EJ, Go AS, Arnett DK, Blaha MJ, et al (2016) Heart Disease and Stroke Statistics-2016 Update: A Report From the American Heart Association. Circulation 133: e38–e360. 10.1161/CIR.0000000000000350 26673558

[2] Marsh JD, Keyrouz SG (2010) Stroke prevention and treatment. J Am Coll Cardiol 56: 683–691. 10.1016/j.jacc.2009.12.072 20723798

[3] Spaander FH, Zinkstok SM, Baharoglu IM, Gensicke H, Polymeris A, et al (2017) Sex Differences and Functional Outcome After Intravenous Thrombolysis. Stroke 48: 699–703. 10.1161/STROKEAHA.116.014739 28143921

[4] Madsen TE, Khoury J, Alwell K, Moomaw CJ, Rademacher E, et al (2017) Sex-specific stroke incidence over time in the Greater Cincinnati/Northern Kentucky Stroke Study. Neurology 89: 990–996. 10.1212/WNL.0000000000004325 28794254 PMC5589794

[5] Brait VH, Jackman KA, Walduck AK, Selemidis S, Diep H, et al (2010) Mechanisms contributing to cerebral infarct size after stroke: gender, reperfusion, T lymphocytes, and Nox2-derived superoxide. J Cereb Blood Flow Metab 30: 1306–1317. 10.1038/jcbfm.2010.14 20145655 PMC2949221

[6] Ong CT, Wong YS, Sung SF, Wu CS, Hsu YC, et al (2017) Sex-related differences in the risk factors for in-hospital mortality and outcomes of ischemic stroke patients in rural areas of Taiwan. PLoS One 12: e185361. 10.1371/journal.pone.0185361 PMC5608367 28934363

[7] Koellhoffer EC, McCullough LD (2013) The effects of estrogen in ischemic stroke. Transl Stroke Res 4: 390–401. 10.1007/s12975-012-0230-5 24323337 PMC4275797

[8] Zaninovich OA, Ramey WL, Walter CM, Dumont TM (2017) Completion of the Circle of Willis Varies by Gender, Age, and Indication for Computed Tomography Angiography. World Neurosurg 106: 953–963. 10.1016/j.wneu.2017.07.084 28736349

[9] Etherton MR, Wu O, Cougo P, Giese AK, Cloonan L, et al (2017) Structural Integrity of Normal Appearing White Matter and Sex-Specific Outcomes After Acute Ischemic Stroke. Stroke 48: 3387–3389. 10.1161/STROKEAHA.117.019258 29127272 PMC5726524

[10] Chauhan A, Moser H, McCullough LD (2017) Sex differences in ischaemic stroke: potential cellular mechanisms. Clin Sci (Lond) 131: 533–552. 28302915 10.1042/CS20160841

[11] Colbert JF, Traystman RJ, Poisson SN, Herson PS, Ginde AA (2016) Sex-Related Differences in the Risk of Hospital-Acquired Sepsis and Pneumonia Post Acute Ischemic Stroke. J Stroke Cerebrovasc Dis 25: 2399–2404. 10.1016/j.jstrokecerebrovasdis.2016.06.008 27363622 PMC5026915

[12] McCullough LD, Mirza MA, Xu Y, Bentivegna K, Steffens EB, et al (2016) Stroke sensitivity in the aged: sex chromosome complement vs. gonadal hormones. Aging (Albany NY) 8: 1432–1441. 27405096 10.18632/aging.100997 PMC4993340

[13] Asdaghi N, Romano JG, Wang K, Ciliberti-Vargas MA, Koch S, et al (2016) Sex Disparities in Ischemic Stroke Care: FL-PR CReSD Study (Florida-Puerto Rico Collaboration to Reduce Stroke Disparities). Stroke 47: 2618–2626. 10.1161/STROKEAHA.116.013059 27553032 PMC5039084

[14] Gibson CL, Attwood L (2016) The impact of gender on stroke pathology and treatment. Neurosci Biobehav Rev 67: 119–124. 10.1016/j.neubiorev.2015.08.020 26657813

[15] Di Carlo A, Lamassa M, Baldereschi M, Pracucci G, Basile AM, et al (2003) Sex differences in the clinical presentation, resource use, and 3-month outcome of acute stroke in Europe: data from a multicenter multinational hospital-based registry. Stroke 34: 1114–1119. 10.1161/01.STR.0000068410.07397.D7 12690218

[16] Morrison HW, Filosa JA (2016) Sex differences in astrocyte and microglia responses immediately following middle cerebral artery occlusion in adult mice. Neuroscience 339: 85–99. 10.1016/j.neuroscience.2016.09.047 27717807 PMC5118180

[17] Nguyen TV, Frye JB, Zbesko JC, Stepanovic K, Hayes M, et al (2016) Multiplex immunoassay characterization and species comparison of inflammation in acute and non-acute ischemic infarcts in human and mouse brain tissue. Acta Neuropathol Commun 4: 100 10.1186/s40478-016-0371-y 27600707 PMC5011964

[18] Tatusova TA, Karsch-Mizrachi I, Ostell JA (1999) Complete genomes in WWW Entrez: data representation and analysis. Bioinformatics 15: 536–543. 10.1093/bioinformatics/15.7.536 10487861

[19] Krug T, Gabriel JP, Taipa R, Fonseca BV, Domingues-Montanari S, et al (2012) TTC7B emerges as a novel risk factor for ischemic stroke through the convergence of several genome-wide approaches. J Cereb Blood Flow Metab 32: 1061–1072. 10.1038/jcbfm.2012.24 22453632 PMC3367223

[20] Tatusova T, DiCuccio M, Badretdin A, Chetvernin V, Nawrocki EP, et al (2016) NCBI prokaryotic genome annotation pipeline. Nucleic Acids Res 44: 6614–6624. 10.1093/nar/gkw569 27342282 PMC5001611

[21] Carvalho BS, Irizarry RA (2010) A framework for oligonucleotide microarray preprocessing. Bioinformatics 26: 2363–2367. 10.1093/bioinformatics/btq431 20688976 PMC2944196

[22] Ritchie ME, Phipson B, Wu D, Hu Y, Law CW, et al (2015) limma powers differential expression analyses for RNA-sequencing and microarray studies. Nucleic Acids Res 43: e47 10.1093/nar/gkv007 25605792 PMC4402510

[23] Ashburner M, Ball CA, Blake JA, Botstein D, Butler H, et al (2000) Gene ontology: tool for the unification of biology. The Gene Ontology Consortium. Nat Genet 25: 25–29. 10.1038/75556 10802651 PMC3037419

[24] Szklarczyk D, Morris JH, Cook H, Kuhn M, Wyder S, et al (2017) The STRING database in 2017: quality-controlled protein-protein association networks, made broadly accessible. Nucleic Acids Res 45: D362–D368. 10.1093/nar/gkw937 27924014 PMC5210637

[25] Rivals I, Personnaz L, Taing L, Potier MC (2007) Enrichment or depletion of a GO category within a class of genes: which test? Bioinformatics 23: 401–407. 10.1093/bioinformatics/btl633 17182697

[26] Glickman ME, Rao SR, Schultz MR (2014) False discovery rate control is a recommended alternative to Bonferroni-type adjustments in health studies. J Clin Epidemiol 67: 850–857. 10.1016/j.jclinepi.2014.03.012 24831050

[27] Shannon P, Markiel A, Ozier O, Baliga NS, Wang JT, et al (2003) Cytoscape: a software environment for integrated models of biomolecular interaction networks. Genome Res 13: 2498–2504. 10.1101/gr.1239303 14597658 PMC403769

[28] Bader GD, Hogue CW (2003) An automated method for finding molecular complexes in large protein interaction networks. BMC Bioinformatics 4: 2 10.1186/1471-2105-4-2 12525261 PMC149346

[29] Tang Y, Li M, Wang J, Pan Y, Wu FX (2015) CytoNCA: a cytoscape plugin for centrality analysis and evaluation of protein interaction networks. Biosystems 127: 67–72. 10.1016/j.biosystems.2014.11.005 25451770

[30] Huang DW, Sherman BT, Lempicki RA (2009) Systematic and integrative analysis of large gene lists using DAVID bioinformatics resources. Nat Protoc 4: 44–57. 10.1038/nprot.2008.211 19131956

[31] Black M, Wang W, Wang W (2015) Ischemic Stroke: From Next Generation Sequencing and GWAS to Community Genomics? OMICS 19: 451–460. 10.1089/omi.2015.0083 26230531

[32] Ye Z, Zhang H, Sun L, Cai H, Hao Y, et al (2018) GWAS-Supported CRP Gene Polymorphisms and Functional Outcome of Large Artery Atherosclerotic Stroke in Han Chinese. Neuromolecular Med. 10.1007/s12017-018-8485-y 29556980

[33] Tan X, Zhang X, Pan L, Tian X, Dong P (2017) Identification of Key Pathways and Genes in Advanced Coronary Atherosclerosis Using Bioinformatics Analysis. Biomed Res Int 2017: 4323496 10.1155/2017/4323496 29226137 PMC5684517

[34] Fahmi RM, Elsaid AF (2016) Infarction Size, Interleukin-6, and Their Interaction Are Predictors of Short-Term Stroke Outcome in Young Egyptian Adults. J Stroke Cerebrovasc Dis 25: 2475–2481. 10.1016/j.jstrokecerebrovasdis.2016.06.021 27402591

[35] Srivastava MV, Bhasin A, Chaudhry R, Sharma S, Subbaiah V, et al (2014) Novel inflammatory biomarkers and their correlation to Chlamydia pneumoniae titres in acute ischemic stroke. J Stroke Cerebrovasc Dis 23: 2391–2396. 10.1016/j.jstrokecerebrovasdis.2014.05.016 25263435

[36] Jin G (2017) The relationship between serum CXCL16 level and carotid vulnerable plaque in patients with ischemic stroke. Eur Rev Med Pharmacol Sci 21: 3911–3915. 28975971

[37] Feng J, Liu YH, Yang QD, Zhu ZH, Xia K, et al (2013) TNFSF4 gene polymorphism rs3861950 but not rs3850641 is associated with the risk of cerebral infarction in a Chinese population. J Thromb Thrombolysis 36: 307–313. 10.1007/s11239-012-0849-9 23184501

[38] Li WZ, Gao CY, He WL, Zhang HM (2016) Association of the interleukin-10 gene -1082A/G genetic polymorphism with risk of ischemic stroke in a Chinese population. Genet Mol Res 15. 10.4238/gmr.15017541 27051029

[39] Banerjee A, Wang J, Bodhankar S, Vandenbark AA, Murphy SJ, et al (2013) Phenotypic changes in immune cell subsets reflect increased infarct volume in male vs. female mice. Transl Stroke Res 4: 554–563. 10.1007/s12975-013-0268-z 24187596 PMC3811047

[40] Bodhankar S, Lapato A, Chen Y, Vandenbark AA, Saugstad JA, et al (2015) Role for microglia in sex differences after ischemic stroke: importance of M2. Metab Brain Dis 30: 1515–1529. 10.1007/s11011-015-9714-9 26246072 PMC4644102

[41] Li Y, Suo L, Liu Y, Li H, Xue W (2017) Protective effects of ginsenoside Rg1 against oxygen-glucose-deprivation-induced apoptosis in neural stem cells. J Neurol Sci 373: 107–112. 10.1016/j.jns.2016.12.036 28131165

[42] Yang JL, Mukda S, Chen SD (2018) Diverse roles of mitochondria in ischemic stroke. Redox Biol 16: 263–275. 10.1016/j.redox.2018.03.002 29549824 PMC5854930

