# Supplementary figures and images for: Sex-specific and opposite modulatory aspects revealed by PPI network and pathway analysis of ischemic stroke in humans
Source: PLoS One. 2020 Jan 3;15(1):e0227481. doi: 10.1371/journal.pone.0227481 (PMC6941802; doi:10.1371/journal.pone.0227481)

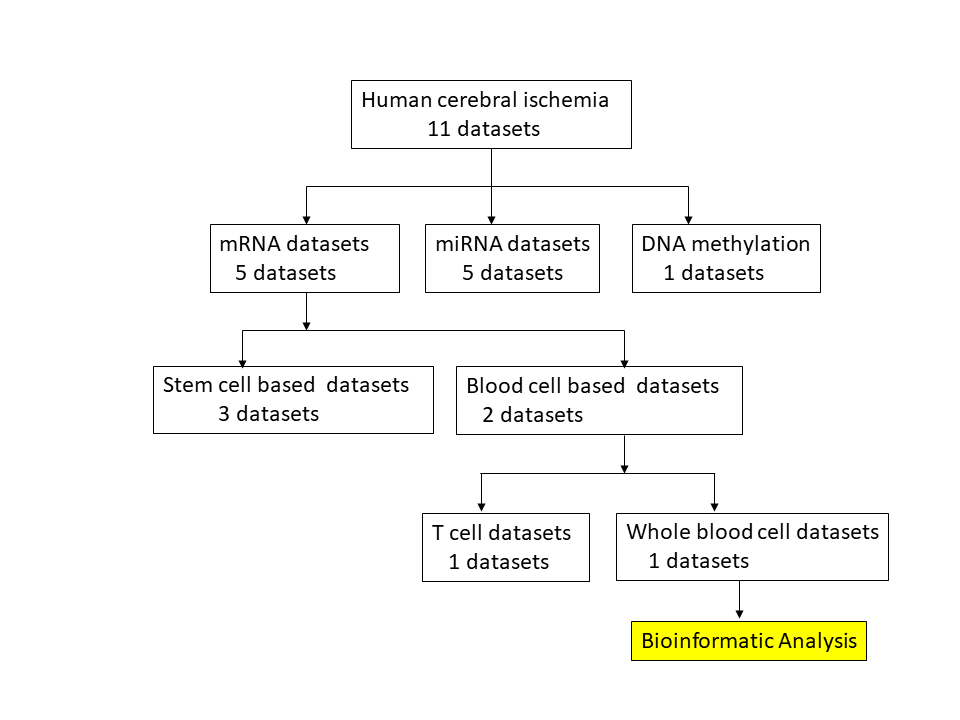

Supplement: S1 Fig — (TIF) [file pone.0227481.s001.tif]

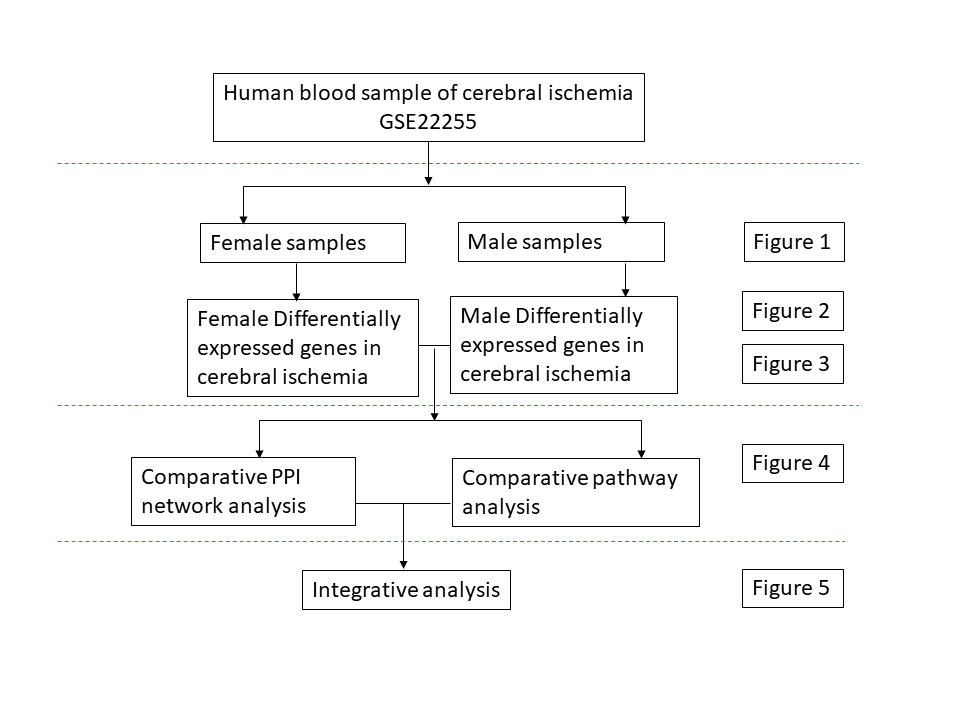

Supplement: S2 Fig — (TIF) [file pone.0227481.s002.tif]
